# Primary Care Clinician and Child Characteristics Impacting Autism Surveillance

**DOI:** 10.3390/brainsci13010018

**Published:** 2022-12-22

**Authors:** Lashae N. Williams, Andrea Trubanova Wieckowski, Mary F. S. Dieckhaus, Yael G. Dai, Fengqing Zhang, Thyde Dumont-Mathieu, Marianne Barton, Deborah Fein, Diana L. Robins

**Affiliations:** 1 A.J. Drexel Autism Institute, Drexel University, Philadelphia, PA 19104, USA; 2 Department of Psychological Sciences, University of Connecticut, Storrs, CT 06269, USA; 3 Department of Psychological and Brain Sciences, Drexel University, Philadelphia, PA 19104, USA; 4 Department of Pediatrics, University of Connecticut School of Medicine, Farmington, CT 06032, USA

**Keywords:** autism, detection, disparities, primary care

## Abstract

Early detection of autism provides access to early intervention and subsequently fewer lifelong challenges. However, disparities in screening have been associated with socioeconomic status (SES) and race, and disparities in surveillance have been associated with clinician knowledge and beliefs about autism identification. The present study examines associations between demographic variables and clinician beliefs, and agreement between screening results and clinician surveillance. Surveillance included activities used by the primary care clinicians (PCCs) to assess risk for autism. PCCs reported their beliefs about autism screening and identification, their sex, race, years in practice, and racial distribution of their patient population. Children’s demographic information was also collected. PCCs identified children as having, or not having, an increased likelihood of autism, and parents of children completed an autism screener. Agreement between screening and surveillance results were examined across PCC, practice, and child demographics. Higher confidence in autism knowledge and screening resources, female PCC sex, and majority White practice patient demographics all predicted agreement between screening and surveillance. Female child sex and higher maternal education also predicted agreement between screening and surveillance. These findings highlight the importance of PCC screening beliefs and child and PCC demographics on the autism identification process.

## 1. A Note on Terminology

In this paper, we make an earnest attempt to use language that is inclusive with the understanding that there is not universal agreement on what terminology is considered inclusive. Many autistic self-advocates have stated their preference for the use of identity-first language [1,2]. In line with the requests of self-advocates and in attempts to present concise language where generalizations are necessary, we use “autistic” throughout this paper, and avoid the medical term autism spectrum disorder, or ASD. There is similar debate regarding best language practices used to describe various racial identities. The present study uses race as a variable in analysis. It should be noted that the variable of race is a proxy for the treatment of individuals based on race, whether the impact of such treatment is privilege or marginalization. Similarly, we use “minoritized” at times to highlight the impact of systemic racism on Black people. Above all, we honor individuals who may prefer different terminology to describe their identities and urge the reader to respect the preferences of individuals of various identities. 

## 2. Introduction

Early diagnosis of autistic children and subsequent early intervention is associated with gains in skills (e.g., intellectual functioning, language use, and adaptive skills) and reduced parental stress [3,4,5,6,7,8,9,10,11,12,13,14,15]. Although autism can be detected as early as 14 months [16,17,18,19], most children in the United States are diagnosed after their fourth birthday, and age of diagnosis is later for children of underrepresented racial and ethnic backgrounds [20]. The American Academy of Pediatrics recommends routine surveillance and screening by Primary Care Clinicians (PCCs) to identify children at increased likelihood of developmental delays, and autism specifically [21,22]. PCCs include pediatricians, nurse practitioners, physician assistants, and any other medical professionals who see children as clinicians for their well child care visits. Surveillance integrates six components: eliciting parent’s concerns; obtaining a developmental history; observing the child; identifying risks, strengths, and protective factors; maintaining a record; and sharing opinions and findings [23]. However, family, PCC, and practice characteristics may influence the effectiveness of both surveillance and screening. 

Family characteristics have been demonstrated to influence the effectiveness of autism identification including accuracy of screening outcomes and PCC concern. For example, caregivers who have (a) less familiarity with early symptoms of autism, (b) perceived or experienced stigma, (c) distrust of healthcare PCCs, and/or (d) lower socioeconomic status (SES) may have lower likelihood of seeking medical services [24,25,26,27,28]. Therefore, these caregivers may miss the opportunity to complete a screener or opt not to report symptoms on an autism screener [24,25]. This has been particularly documented for Black families, who report greater distrust of healthcare PCCs and who under-report autism-specific symptoms in their children, relative to families of other racial backgrounds [24,25]. At least one study demonstrated that parental endorsement of symptoms differs among racial and ethnic groups [25], which can affect outcome on parent-report screeners like the Modified Checklist for Toddlers, Revised with Follow-Up (M-CHAT-R/F) [29]. Donohue and colleagues [25] found that Black caregivers reported fewer concerns about social engagement and restricted and repetitive behaviors relative to White caregivers, even though Black children in the sample had more severe social impairment. Additionally, patient race is associated with when and how often physicians make referrals to specialists for developmental evaluations, with children of color receiving delayed referrals for developmental evaluations [26]. SES is also associated with screening accuracy [30], age of evaluation for autism [31,32,33,34], and access to services [35]. Lower maternal education, often used as a marker of SES, is associated with more frequent positive screens on initial M-CHAT(-R), and a greater likelihood of reverting to screen negative after structured Follow-Up is administered. This pattern suggests that caregivers of lower SES background may be less aware about clinically significant symptoms [30], or that their perspectives of symptoms are differing and absent from samples used to validate screening measures.

Disparities in detection of autism may also stem from PCC experience, beliefs, and demographic characteristics. In a systematic review, McCormack and colleagues [36] reported that many PCCs demonstrated inadequate, or in some cases inaccurate, knowledge about the etiology and symptoms of autism. Similarly, in a study of 114 PCCs across the U.S., more than half of the PCCs expressed a desire for more specialized training in autism [37]. Many PCCs do not feel confident in their abilities to screen for autism, manage challenging behaviors associated with autism, provide resources to families, and communicate about treatment options with families [37,38]. 

In addition to knowledge of autism, personal beliefs may affect PCC engagement in autism surveillance. Physician-held prejudices and stereotypes may affect responses to concerns about autism from patients of color [39]. For example, some clinicians hold biases about the prevalence of certain disorders in populations of color, which may explain the reason that Black children are often misdiagnosed with conduct disorder or disruptive disorders prior to receiving an accurate diagnosis for autism [39,40]. PCCs are less likely to screen for autism if families are Hispanic or of lower SES [41]. Some PCCs also believe that White families who reside in urban or suburban settings will react more positively to an autism diagnosis relative to non-White families living in rural locations [42]. These impressions may affect PCC behavior. A physician’s level of multicultural competency and experience working with different racial and ethnic groups may positively or negatively affect disparities in detection of autism, and a physician’s behavior during an appointment can directly affect a family’s sense of trust and willingness to engage with the healthcare system [24]. 

Moreover, PCC and practice characteristics may influence the likelihood and accuracy of autism surveillance. For example, personal experiences with autism, education about autism, and amount of clinical experience relate to PCC knowledge about autism [36]. PCC demographic characteristics, and the way that these factors interact with family characteristics, may be associated with how PCCs conduct autism surveillance. Male PCCs self-reported over-diagnosing autism relative to female PCCs [42]. PCC-reported reasons for overdiagnosis include assisting families in accessing academic services or resources through health insurance. Finally, practice-level demands (e.g., appointment length), lack of autism-specific trainings for employees, and insufficient office-based systems to support referrals, billing, and monitoring outcome, serve as barriers to screening and referrals [14,37,43,44]. PCCs in the Northeastern United States report over-diagnosing autism more than PCCs in the Western United States [42], which suggests that geographic location of the practice can affect whether a diagnosis is made. Similarly, PCCs located in non-academic medical centers reported greater overdiagnosis than in other locations [42].

Universal standardized screening promotes earlier identification of children with high-likelihood of autism, and reduces the delay in diagnosis for children from diverse and lower SES families [34,45]. Indeed, screening is associated with reduced delay in diagnosis above and beyond child and family factors [46]. PCC agreement with screening can affect PCC decision-making, such as whether or not to refer a child identified at increased likelihood of autism for diagnostic evaluation [47]. However, the impact of family, PCC, and practice characteristics on the accuracy of routine screening and surveillance is not well understood. 

The current study examined how factors at the child and family, PCC, and practice levels relate to the agreement between PCCs’ judgment of concern for autism through surveillance and a child’s screening result. Child and family characteristics included race, sex, and maternal education. PCC variables included race, sex, years in practice, confidence in autism knowledge, beliefs about screening resources, and beliefs about the utility of formal autism screening. Practice characteristics were patient demographics of children seen in their practice. Agreement has significant implications for families’ participation in the diagnostic process. To our knowledge, this is the first large-scale study to simultaneously explore child, PCC, and practice characteristics that influence the agreement between routine screening and PCC surveillance. We hypothesized that there will be less agreement between surveillance and screening outcome in PCCs with less confidence about their autism knowledge, those with beliefs about inadequate resources for surveillance, and those who value the use of formal autism-specific screening less. We also hypothesized that there will be less agreement when PCCs are male, White, have fewer years in practice, and work with a patient demographic that is primarily comprised of minoritized individuals. Finally, we hypothesized that there will be less agreement between surveillance and screening outcome amongst male children, those with lower maternal education, and Black children.

## 3. Materials and Methods

### 3.1. Participants

One hundred and twenty-nine PCCs, across 28 primary care practices, were recruited across three sites (Connecticut, Pennsylvania, and Georgia) to screen toddlers for autism during primary care check-ups from 2013 to 2019. Pediatric PCCs (92 female, 35 male, 2 PCCs who did not identify their sex; 25 racial or ethnic minority) in these practices had an average of 16.6 years (*SD* = 4.9 years) of experience. PCCs consisted of 87 pediatricians, 7 family medicine physicians, 26 nurse practitioners, 5 physician assistants, 2 social workers, and 1 public health practitioner; one PCC did not specify specific training background. Practices reported the racial demographic of their patients, with 9 practices reporting their patient population comprising primarily racially minoritized individuals. 

Children were screened for autism at 12 months, 15 months, or 18 months of age, based on randomization at the practice level. Children enrolled at 12 months or 15 months were rescreened at 18 months; all children were rescreened at 24 and/or 36 months. Demographic information, including race and maternal education, a measure of socioeconomic status, was collected from parents of enrolled children. The parent study from which our data was collected screened 5961 toddlers at least once across daycares and pediatric practices. The present study was limited to children of Black or White race (N = 4990); small sample sizes precluded inclusion of other races. After removing missing data of children across practices (2 were missing practice and PCC information, 479 were missing race, 272 were missing maternal education, 94 were missing sex), our remaining sample consists of 4143 children screened across 28 practices (see Table 1 for demographic information of enrolled children). 

### 3.2. Measures

#### 3.2.1. Screening

Infant Toddler Checklist (ITC) [48]. ITC is a 24-item questionnaire designed to detect communication and social delays in children 6–24 months old. Scores below the 10th percentile indicate increased likelihood of autism. Internal consistency is high (0.86–0.92), sensitivity for autism is high (0.93), but specificity is low, since it is designed to detect broader communication delays [49]. Based on findings that ITC detects autism at 12 months in a general pediatric sample [50], ITC was administered during the 12-month well-child care visits.

First Year Inventory Lite (FYI-L) [51]. FYI-L is an autism-screener for children 12 months of age. The FYI-L consists of the 20 FYI items with the highest item-domain correlations. It is highly correlated with the original FYI score. Sensitivity = 0.44, specificity = 0.99, PPV = 0.14–0.31 and NPV = 0.99 [52]. The FYI-L was administered during the 12-month and 15-month well-child care visits. 

Modified Checklist for Autism in Toddlers, Revised, with Follow-Up [53]. M-CHAT-R/F is a 20 item, autism-specific tool for toddlers 16–30 months old. A score greater than or equal to 3 is considered positive for greater likelihood of autism. Children in the moderate likelihood range (score 3–7) receive the structured M-CHAT-R Follow-up interview, which ascertains additional detail and examples confirming or disconfirming autistic behaviors. The Follow-up interview is positive when the total score is ≥2. M-CHAT-R/F was administered at 15-month, 18-month, 24-month, and 36-month well-child care visits. Sensitivity = 0.85, specificity = 0.99, PPV = 0.48, and NPV = 0.99 [53]. 

#### 3.2.2. PCC Concern

During each well-child care visit, PCCs were asked to indicate whether they had a concern regarding autism and if so, to indicate the type of concern, including language concerns, social engagement concerns, restrictive or unusual behavior concerns, or other concerns. For each of the categories, the PCCs were also asked to indicate what information led to their concern and were able to check all that applied from following responses: observation, surveillance over multiple visits, family history, screening results, parent concern, or parent report. Although not required to do so, the PCCs had the option to view the screening results before indicating their concerns; as such, they had the opportunity to add in the screening results to their surveillance tasks in determining their level of concern. 

#### 3.2.3. PCC Characteristics and Beliefs Inventory

All PCCs completed a version of the Screening for Developmental Delay in Young Children, A National Survey [54], adapted with permission from the author (personal communication). The inventory includes questions regarding developmental screening beliefs and practices. All questions were presented on a Likert scale from 1 (i.e., Strongly Disagree or Very Unlikely) to 5 (i.e., Strongly Agree or Very Likely). Three variables (i.e., PCC confidence, beliefs about resources, and likeliness to use formal autism screening) were created based on an average of ratings of relevant questions for those constructs (See Table 2). For confidence, PCCs ranked their confidence identifying autism, communicating with families about autism, and making appropriate referrals (range = 2.17–4.00). For resource beliefs, PCCs ranked their beliefs about having sufficient time and reimbursement to conduct screening (range = 2.10–4.25). For formal screening beliefs, PCCs ranked how likely they were to use a formal autism screener, such as the M-CHAT (range = 2.00–4.67). In addition, the questionnaire included questions regarding the physician characteristics, including PCC type (e.g., pediatrician, family practice physician, or nurse practitioner), length in practice post residency, age, sex, and race.

#### 3.2.4. Practice Characteristics

Each practice completed a questionnaire regarding their practice characteristics, informed by the Screening for Developmental Delay in Young Children, A national Survey [54]. The questionnaire solicited information about the patient demographic, including the percentage of the children seen in the practice from each racial background (see Participants). 

### 3.3. Procedures

The current study is a cross-sectional, experimental design. PCCs completed the Screening for Developmental Delay in Young Children survey at the time of enrollment into the study. Similarly, each participating practice, completed the survey regarding practice characteristics at the time of enrollment. Parents completed the ITC, FYI-L, and/or M-CHAT-R/F during well-child visits, and PCCs separately identified children they believed to be at increased likelihood of autism during these visits based on surveillance. 

All enrolled PCCs and children consented to participate in the study. Research was approved and overseen by the Institutional Review Board (IRB) of each participating university. Original research records are kept in locked cabinets in a locked room on a secured floor. Practice, PCC, and child data used in the present study were de-identified to protect anonymity. 

### 3.4. Analyses

Logistic regression was conducted to examine factors significantly related to agreement or disagreement. All analyses were conducted in RStudio Version 1.3.1093 [55]. PCC Beliefs about resources and use of formal autism screening, as well as confidence in providing care to autistic patients were explored in relation to agreement between screening outcome and PCC indicated concern. Screening outcome and PCC concern were both dichotomized as high likelihood of autism vs. low likelihood of autism. Child race, SES, and sex were explored in relation to agreement between screening outcome and PCC indicated concern to determine associations between child factors and disparity in effective autism screening and surveillance. To determine what PCC and practice variables are associated with disparity in effective autism screening and surveillance, physician self-efficacy, attitudes about screening and diagnosis, sex, time in practice, practice location, and demographics of families served were explored in relation to screening-surveillance agreement. A binary agreement variable was created where “1” indicated agreement between a PCC’s concern, or lack of concern, for autism and the child’s screening outcome on the FYI, ITC, and/or M-CHAT-R/F (i.e., high-likelihood/screen positive or low-likelihood/screen negative). A code of “0” indicated lack of agreement between PCC concern and screening outcome. If a child was positive on a screener at any timepoint, the child was considered high-likelihood. Similarly, if a PCC indicated autism concern for a child at any timepoint, the child was considered to have PCC concerns. 

ROSE resampling [56] was conducted to account for the imbalance between the number of participants in the agreement vs. disagreement groups driven largely by children classified as true negative which were those with low-likelihood for autism and no PCC concern. The efficiency of analyses employing resampling techniques is typically assessed using accuracy metrics to compare training and test models, where the statistic for the test model is reported. Given the nature of our data, the class distributions in our sample were heavily imbalanced and weighted towards true negative results. The *F*1 statistic combines positive predictive value and sensitivity while accounting for imbalances driven by true negatives. As such, both *F*1 and prediction accuracy were reported for evaluating model performance. Similar to other accuracy metrics, *F*1 ranges from 0–1, where higher values are indicative of better model performance. Values greater than 0.50 indicate more accurate classification of the logistic model in the test set compared to the training set.

## 4. Results

Of the 4143 children in our sample, 3637 (88%) demonstrated agreement between PCC surveillance (+/− screening results) and screening result. Of those in agreement, 3543 children (97%) had no concern for autism at all, comprising the majority of our sample. Table 3 shows the distribution of PCC concern (which may have been informed by screening results) and screening outcome, before resampling. 

### 4.1. PCC Beliefs

PCC beliefs (i.e., confidence, resources, screening beliefs) were entered into a logistic regression model to determine which factors predicted increased likelihood of agreement between PCC concern and child screening outcome (See Table 4). The overall model was significant (*F*1 = 0.70, Accuracy = 0.56, 95% CI = 0.53–0.59). Higher PCC confidence (β = 0.50, *p* = 0.002) significantly predicted a greater likelihood of agreement between PCC concern and screening outcome. Higher reported PCC resources also significantly predicted agreement (β = 0.43, *p* = 0.01). PCC beliefs about the use of formal autism screening did not significantly increase the likelihood of agreement.

### 4.2. PCC/Practice Characteristics

PCC characteristics (i.e., race, sex, years in practice) and practice characteristics (i.e., patient demographics) were entered into a logistic regression model to determine which factors increased likelihood of agreement between PCC concern and child screening outcome (See Table 5). The model was significant (*F*1 = 0.68, Accuracy = 0.45, 95% CI = 0.42–0.48). PCC sex significantly predicted agreement between PCC concern and screening outcome (β = −2.46, *p* < 0.001), with female PCCs demonstrating more agreement than male PCCs. The racial demographic of the practice’s patients significantly predicted agreement between PCC concern and screening outcome. Specifically, increased likelihood of agreement between PCC concern and screening outcome was predicted by practices who served majority White patients compared to those who see an equal proportion of patients from majority and minoritized racial backgrounds (β = −0.89, *p* < 0.001) or primarily minoritized patients (β = −0.47, *p* = 0.001). Agreement was also predicted by practices who served an equal racial proportion compared to primarily minoritized patients (β = −2.28, *p* < 0.001). Neither PCCs’ years in practice, nor the race of PCCs predicted likelihood of agreement. 

### 4.3. Child Characteristics

Child characteristics (i.e., race, sex, and maternal education) were entered into a logistic regression to determine factors predicting likelihood of agreement between PCC concern and a child’s ultimate diagnosis (i.e., autism or non-autism; See Table 6). The full model was significant (*F*1 = 0.75, Accuracy = 0.63, 95% CI = 0.60–0.66). Child sex and maternal education both significantly predicted agreement between PCC concern and diagnostic outcome. Specifically, female children were more likely to demonstrate agreement between PCC concern and diagnostic outcome than males (β = 0.44, *p* < 0.001). Additionally, children whose mothers had bachelor’s degrees were more likely to have agreement between PCC concern (β = −0.70, *p* < 0.001) and screening result compared to children whose mothers completed fewer years of education, including a high school diploma or less, technical school, or some college. Child race did not significantly predict agreement.

## 5. Discussion

Autism surveillance by pediatric primary care PCCs is an important part of the screening and referral process. Various factors impact how accurate PCCs are in their surveillance. Given the disparities in who receives formal autism screening, referrals, and ultimately diagnosis, it is important to examine PCC and family factors associated with these crucial action steps that impact long-term outcomes for children on the autism spectrum. 

Consistent with our hypothesis, among PCCs who had the opportunity to report autism concerns based on surveillance (including results of standardized screening), those who are more confident in their knowledge and ability to communicate about autism are more likely to have results of autism surveillance to match autism screening outcome. Although some may argue that only positive surveillance *or* positive screening is necessary to inform referrals, families often rely on the expertise of their PCCs to make decisions about seeking further evaluation. Therefore, families are best served when PCCs combine surveillance findings and screening results in determining the appropriateness of making a referral to EI and for diagnosis. Pediatric PCCs are tasked with providing expertise on a broad range of health and developmental outcomes for children. Many PCCs demonstrate a need or express a desire to obtain more training on the signs of autism and how to refer families appropriately [37,38]. These reported gaps in autism knowledge present opportunities to increase autism-specific education during formal healthcare training, in specialized residency programs, or through continuing education requirements for all PCCs. It is also important to acknowledge that PCCs may have worries about incorrectly raising concerns about autism to families, resulting in undue stress or increased burden on diagnostic specialists. Empowering PCCs to confidently identify potential signs of autism and refer families to specialists appropriately, and to confidently communicate concerns to families, may mitigate some of those fears [44,57]. Research also has documented PCCs’ concerns about lacking resources to support screening and surveillance, with one study noting that 85% of PCCs in their sample reported a need for increased time [37]. Our results suggest that these concerns may be related to surveillance outcomes. Ideally, PCCs should have adequate time and receive adequate reimbursement for time spent on surveillance. Although PCCs may have concerns about the utility of formal screening [43], this factor was not associated with screening-surveillance agreement, suggesting that PCCs are able to effectively engage in surveillance despite interference from concerns about screening tools.

PCCs from practices who saw a majority White patient population were more likely to have surveillance that matched screening outcome compared to practices that saw mostly racially minoritized patients, supporting our hypothesis. It is difficult to speculate on the reason for this finding as our analyses did not include interaction terns. However, the decreased agreement observed for the PCCs in our sample serving primarily minoritized children and families could have been driven by differences in parent reporting on screeners [25], PCC bias in surveillance [26], or a combination of both. Similar to previous findings [42], PCC sex was associated with autism identification. Azim and colleagues [42] found that male pediatricians self-reported overdiagnosing autism compared to female pediatricians. The present study did not examine the relationship between screening results and diagnostic outcomes. However, male sex was associated with lower screening-surveillance agreement in our sample of PCCs. Notably, PCCs in this study were predominately female, consistent with documented sex differences in pediatric medical practice [58]. Thus, this effect may be related to differences in socialized characteristics that lead to a dearth of pediatric PCCs. Across sexes, it is important that PCCs continue to receive adequate training on surveillance to ensure best practices. We also found that the number of years a PCC was in practice was not associated with agreement. Taken together with our findings on PCC confidence and increased agreement, effective autism surveillance may be related to PCCs’ autism-specific experiences rather than experience measured by time in practice.

Surveillance classification matched screening results more often for children with higher maternal education compared to fewer years of formal education, consistent with previous research that lower maternal education is associated with inflated screening results [30]. These findings highlight the impact of social determinants of health on autism screening and surveillance [59]. Contrary to our hypothesis, female children in our sample received more accurate surveillance, which is counter-intuitive given the known disparities in the diagnosis of autism in female children. However, this finding may indicate progress towards ameliorating the gender gap in autism detection. Alternatively, it may indicate that both screening and PCC surveillance missed females at increased likelihood of autism in our sample, which is not possible to ascertain without evaluating all of the screen negative and no concern cases. Further research is necessary to understand the explanation for this finding, as our study did not include a diagnostic component. Child race was not associated with likelihood of agreement. Research is necessary to understand the explanation for this finding, as our study did not include a diagnostic component.

## 6. Conclusions

Given potential biases or blind spots in PCCs during surveillance, formal autism screening is important. However, neither screening nor surveillance is perfect. Both strategies should work in tandem to optimize the chance that every autistic child is identified and referred to a diagnostic specialist as young as possible. To improve the accuracy of surveillance, PCCs would benefit from additional training in autism and adequate resources to conduct surveillance. Additionally, greater racial and socioeconomic diversity in the patient populations served by PCCs is related to increased screening-surveillance agreement, highlighting the importance of ensuring that all PCCs gain experience caring for diverse patient populations. PCCs also need continuing education opportunities to work with and learn about diverse populations to ensure that autism surveillance is not unduly impacted by systemic racism and classism, which impedes access to care.

### Limitations

Our study has several limitations. As noted above, the PCCs in this study were not blind to screening results. They had the option to conduct surveillance independently, or to consider screening results as part of their surveillance, to inform their decision to refer a child or not We do not know the proportions that conducted surveillance in isolation versus integration of surveillance and screening in their decision-making. Therefore, this pragmatic study reinforces what is “real world” practice rather than allow for a comparison of surveillance versus screening as related to referral. Future studies interested in measuring outcomes by surveillance versus screening should use a blinded design. The categorization of variables was based on the distribution of sample frequencies. The authors acknowledge that there are multiple ways in which the data could have been characterized for analysis, but the current methods were designed to allow for the most robust analyses given the available data. Similarly, we did not include ethnicity in the analysis and limited the race of the children in our sample to Black and White children, as the number of participants from other racial groups and Latinx children was not substantial enough for our analyses. However, we acknowledge the complex interplay between race and ethnicity and future studies may account for more detailed data collection of these demographic variables. Finally, the present study was not powered to examine how interactions between PCC and child characteristics affected surveillance agreement with screening. Future research may consider examining such interactions to allow for more robust analyses and conclusions.

## Figures and Tables

**Table 1:** Child Demographics.

Characteristics	Male	Female	Total
	*N* (%)	*N* (%)	*N*
Race			
White	1654 (52.1)	1521 (47.9)	3175
Black	473 (48.9)	495 (51.1)	968
Maternal Education			
High School or Less	358 (54.1)	304 (45.9)	662
Technical School/Some College	373 (51.2)	356 (48.8)	729
Bachelor’s Degree or More	1396 (50.7)	1356 (49.3)	2752

**Table 2:** PCC Beliefs.

Characteristics	*M (SD)*
PCC Confidence	3.28 (0.34)
PCC Resources	2.95 (0.39)
Use of Formal Autism Screeners	3.70 (0.48)

Note. PCCs ranked each characteristic on a scale of 1–5, where 1 was “Strongly Disagree” or “Very Unlikely” and 5 was “Strongly Agree” or “Very Likely”.

**Table 3:** Frequencies of PCC Concern and Screening Results.

*n*(*%*)	Positive Screen	Negative Screen	All Screens
PCC Concern	**94 (2%)**	77 (2%)	171 (4%)
No PCC Concern	429 (10%)	**3543 (86%)**	3972 (96%)
Total	523 (12%)	3620 (88%)	4143 (100%)

Note. Bolded cells indicate agreement between PCC concern and screening results.

**Table 4:** H1: PCC Beliefs Regression.

Predictor	Beta	*p*	*F*1
(Intercept)	−2.96 **	0.001	0.703
Confidence	0.499 **	0.002	
Resources	0.431 *	0.010	
Formal Screening	0.026	0.477	

* *p* < 0.05, ** *p* < 0.01.

**Table 5:** H2: PCC and Practice Characteristics Regression.

Predictor	Beta	*p*	*F*1
(Intercept)	0.970	0.000 ***	0.681
Patient Demographics 1: Majority White vs. Equal Majority-Minority	−0.89	0.000 ***	
Patient Demographics 2: Majority White vs. Minoritized	−0.472	0.001 **	
Years in Practice	0.011	0.301	
PCC Sex	−2.28	0.000 ***	
PCC Race	−0.21	0.379	

** *p* < 0.01, *** *p* < 0.001.

**Table 6:** H3: Child Characteristics Regression.

Predictor	Beta	*p*	*F*1
(Intercept)	0.099	0.220	0.754
Child Race	−0.104	0.347	
Child Sex	0.443	0.000 ***	
Maternal Education 1: Bachelor’s vs. Some College	−0.701	0.000 ***	
Maternal Education 2: Bachelor’s vs. High School	−0.655	0.000 ***	

*** *p* < 0.001.

## Data Availability

Data is available at the NIMH data archive, National Database for Autism Research Collection C2666.
